# Auto Machine Learning and Convolutional Neural Network in Diabetes Mellitus Research—The Role of Histopathological Images in Designing and Exploring Experimental Models

**DOI:** 10.3390/biomedicines13061494

**Published:** 2025-06-18

**Authors:** Iulian Tătaru, Simona Moldovanu, Oana-Maria Dragostin, Carmen Lidia Chiţescu, Alexandra-Simona Zamfir, Ionut Dragostin, Liliana Strat, Carmen Lăcrămioara Zamfir

**Affiliations:** 1 Department of Morphofunctional Sciences I, “Grigore T. Popa” University of Medicine and Pharmacy, 700115 Iasi, Romania; iulian-alexandru-i-tataru@d.umfiasi.ro (I.T.); carmen.zamfir@umfiasi.ro (C.L.Z.); 2 Department of Computer Science and Information Technology, Faculty of Automation, Computers, Electrical Engineering and Electronics, “Dunarea de Jos” University of Galati, 800146 Galati, Romania; 3 The Modelling & Simulation Laboratory, “Dunarea de Jos” University of Galati, 47 Domneasca Str., 800008 Galati, Romania; 4 Research Centre in the Medical-Pharmaceutical Field, Faculty of Medicine and Pharmacy, “Dunarea de Jos” University of Galati, 800201 Galati, Romania; carmen.chitescu@ugal.ro (C.L.C.); ionut.dragostin@yahoo.com (I.D.); 5 Department of Medical Sciences III, “Grigore T. Popa” University of Medicine and Pharmacy, 700115 Iasi, Romania; simona-zamfir@umfiasi.ro; 6 Department of Mother & Child, “Grigore T. Popa” University of Medicine and Pharmacy, 700115 Iasi, Romania; liliana.strat@umfiasi.ro

**Keywords:** histopathological image, PyCaret Auto Machine Learning, custom-built convolutional neural network, diabetes mellitus

## Abstract

Histopathological images represent a valuable data source for pathologists, who can provide clinicians with essential landmarks for complex pathologies. The development of sophisticated computational models for histopathological images has received significant attention in recent years, but most of them rely on free datasets. **Materials and Methods:** Motivated by this drawback, the authors created an original histopathological image dataset that resulted from an animal experimental model, acquiring images from normal female rats/rats with experimentally induced diabetes mellitus (DM)/rats who received an antidiabetic therapy with a synthetic compound (AD_SC). Images were acquired from vaginal, uterine, and ovarian samples from both MD and AD_DC specimens. The experiment received the approval of the Medical Ethics Committee of the “Gr. T. Popa” University of Medicine and Pharmacy, Iași, Romania (Approval No. 169/22.03.2022). The novelty of the study consists of the following aspects. The first is the use of a diabetes-induced animal model to evaluate the impact of an antidiabetic therapy with a synthetic compound in female rats, focusing on three distinct organs of the reproductive system (vagina, ovary, and uterus), to provide a more comprehensive understanding of how diabetes affects female reproductive health as a whole. The second comprises image classification with a custom-built convolutional neural network (CB-CNN), the extraction of textural features (contrast, entropy, energy, and homogeneity), and their classification with PyCaret Auto Machine Learning (AutoML). **Results:** Experimental findings indicate that uterine tissue, both for MD and AD_DC, can be diagnosed with an accuracy of 94.5% and 85.8%, respectively. The Linear Discriminant Analysis (LDA) classifier features indicate a high accuracy of 86.3% when supplied with features extracted from vaginal tissue. **Conclusions:** Our research underscores the efficacy of classifying with two AI algorithms, CNN and machine learning.

## 1. Introduction

The growing prevalence of diabetes mellitus represents a medical problem of maximum interest [1]. All the renal, vascular, cardiac, ophthalmic, and nervous diabetes mellitus complications are well known and widely debated. At the same time, however, there are more and more discussions about the relevance of gender in diabetes mellitus, under the conditions in which, especially in women, diabetes generates diverse sexual dysfunctions and reproductive problems [2]. How much female reproductive function can be affected by diabetes and what mechanisms are involved are just two of the questions that arouse interest today. In the same context, the discovery of novel therapeutic options for diabetes mellitus supports the interest in developing this topic. The application of advanced computational techniques, particularly deep learning (DL) and Machine Learning (ML), to improve the classification accuracy of genital organs has gained significant popularity and attention in the last decade. DL and ML are two subsets of artificial intelligence (AI), with excellent applicability in various medical imaging tasks, such as tumor segmentation or categorization, anatomical contour delineation, therapy outcome prediction, feature extraction, and classification. DL models applied in gynecological pathologies can enhance clinicians’ ability to categorize and diagnose different cases with increased reliability and consistency using in parallel the features extracted automatically from medical images of various image types, mainly histopathology images [3].

DL and ML have attracted more and more attention in recent years for their ability to improve diagnostic accuracy and clinical decision-making across a wide range of medical imaging applications.

In new research, the authors looked at how well AI algorithms could classify histopathological images of the uterus [4,5,6,7,8,9,10,11,12,13], the vagina [14,15,16,17], and the ovaries [18,19,20,21,22,23,24]. Many different types of CNNs were used, including pre-trained ones [4,5,7,12,13,16,19,20,21,22,23,24], custom-built CNNs [8,9,10,14], CNNs and ML and DNNs for classifying morphological and geometric features [14,15], and a mix of a CNN and ML with classifier algorithms replacing the Softmax function [13,17,18].

This research emphasizes the growing prevalence and viability of DL and ML in enhancing the classification accuracy of female reproductive organs. The novelty consists of studying three organs from the female reproductive system (the vagina, ovaries, and uterus). The histopathological images were analyzed with texture features (contrast, entropy, energy, and homogeneity) extracted from the co-occurrence matrix [25]; these features represent the input into PyCaret AutoML [26,27]. Using open-source AutoML tools ensures the employment of 15 ML classifiers and the hyperparameter fine-tuning process. Additionally, custom-built CNNs were applied in the classification process. Although the authors [4,5,7,12,13,16,19,20,21,22,23,24] reported the best results in classifying histopathological images using pre-trained CNNs, the classification accuracy was very low on the proposed dataset.

The following parts of this work are organized as follows. Section 2 highlights the published literature, identifies the research gap, and outlines the authors’ contributions. Section 3 outlines the materials and methods employed, encompassing the hardware, software, dataset, and AI algorithms utilized. Section 4 presents the results, assessment, and comparison to state-of-the-art methods. Section 5 closes the study with suggested insights for further research.

## 2. Literature Review

Considered not only a major global public health problem, but also a specific and very complex pathological condition, diabetes mellitus is far from being just a metabolic disorder whose defining aspects have all been identified, as there are still aspects yet to be explored.

The consequences of diabetes mellitus on female reproductive function have often been underestimated, as its impact is not perceived to be as severe as that on renal, neurological, ophthalmic, and cardiovascular health [28,29]. Diabetes mellitus causes numerous cellular and molecular changes in the components of the female reproductive system. Once it develops, it triggers the onset of a complex and often silent pathology, especially in comparison with other affected systems, but leads to gradual and significant impairment of reproductive function [30].

Glucose homeostasis and insulin secretion require precise and highly refined regulatory mechanisms [29]. Many factors have the potential to affect these mechanisms, generating marked gender differences in the evolution and complications of diabetes. Recent data claim that the long-term effects of diabetes mellitus on women of reproductive age and menopausal and postmenopausal women are of major significance. The disturbances in these regulatory mechanisms underlie female reproductive dysfunctions; however, due to their variability, providing a comprehensive explanation for the majority of these issues remains difficult [31,32].

Diabetic women are at an increased risk of vaginal and recurrent urinary tract infections, infertility, menstrual cycle impairment, sexual dysfunctions, and severe pregnancy-related complications (pregestational or gestational diabetes affecting the maternal and embryonic organism) [33]. Polycystic ovary syndrome (PCOS), due to its connection to insulin resistance, may also generate dysfunctions in female reproductive function. All of these aspects require a very thorough exploration that takes into account the structural complexity of the female genital system and the multiple and overwhelming hormonal interferences.

Changes in diabetes-induced vaginal, uterine, and ovarian morphology may be explained up to a point, but the mechanisms involved require further investigation. Diabetes induces changes in the normal morphology of female reproductive organs, closely correlated with a sharp decrease in their functions. Advanced vaginal fibrosis, follicular degeneration, an increase in the number of atretic ovarian follicles, and uterine mucosa atrophy can reduce or even compromise female reproductive functions.

In vivo experimental models are utilized to gain a deeper understanding of these processes, offering valuable insights when extrapolated to humans [34]. With proper design, these animal models can provide numerous data related to morphological changes in the female reproductive system, the underlying mechanisms that support it, and new targeted therapies [35].

### 2.1. Research Gap and Contribution

Despite the successful application of DL in the classification of histopathological images, there is an urgent requirement for strong and reliable systems particularly designed to classify the gynecologic tissues in the case of diabetes mellitus. Current research predominantly focuses on the features of individual DL models or pre-trained CNNs, which are time-consuming, may fail to encompass the full complexity of medical images, and may not fulfill the requisite standards of accuracy and dependability for clinical applications.

By integrating two AI algorithms, an optimized CNN model and AutoML, the classification of images into healthy and gynecological pathologies significantly improved the accuracy that was obtained and became complete.

Table 1 presents state-of-the-art studies that employed DL or ML models for the classification of genital pathology. The studies were analyzed in concordance with the type of tissue analyzed, their weakness, and the AI algorithm used. For a reliable comparison, we only used studies where histopathological images were selected.

The common weakness of studies in the scientific literature is that these deal with only one tissue type (uterine [2,3,4,5,6,7,8,9,10,11,12,13], vaginal [14,15,16,17], or ovarian [18,19,20,21,22,23,24]). Only Onishi et al. [13] proposed to analyze vaginal and uterine tissue. The majority of studies used DNN algorithms, and the extraction of features from histopathological images was proposed by Volinsky-Fremond et al. [14]. When the use of morphological and genomic features was proposed, Jeleń et al. [15] **used** geometric features that fed a neural network (NN) and a support vector machine (SVM). A recent study by Zafar et al. [11] highly correlated 65 features in a classification process with an NN.

The hybrid models that used pre-trained CNNs and MLs were depicted by Rajan et al. [17], where the Softmax function was replaced with extreme gradient boost (XGBoost), SVM, k-nearest neighbor (KNN), InceptionV3, and residual networks (ResNet). Zhou et al. [18] proposed SVM, random forest (RF), XGBoost, and a custom-built CNN. Wang et al. [20] focused on the pre-trained CNN InceptionV3 and Vision Transformer (ViT).

### 2.2. Novelty and Contribution

By combining the advantages of custom-built architectures and PyCaret AutoML, our study fills a research gap because the time-consuming, low-classification-accuracy use of only an AI algorithm can be avoided. The contributions of this study are as follows:

1. The acquisition of four tissue types (healthy, uterine, vaginal, and ovarian) and the creation of a histopathological image dataset for both MD and AD_DC specimens;
2. Building, optimizing, and training a custom-built CNN with four layers;
3. Extracting second-order features (contrast, entropy, energy, and homogeneity) from the co-occurrence matrix;
4. Training the end-to-end PyCaret AutoML algorithm with correlation, energy, contrast, and homogeneity textural features;
5. A performance evaluation of both the CNN and AutoML in terms of accuracy, F1-score, and area under the curve (AUC).

## 3. Materials and Methods

The experiments described in this article are part of a larger experiment, which was extensively detailed in a separate article [36] (https://doi.org/10.3390/biomedicines13040922).

### 3.1. Microscope

Samples from all female rats (n = 7/group) from the control, (untreated)/induced, untreated diabetes mellitus, and induced diabetes mellitus treated with a synthetic compound groups were collected, fixed in 4% formaldehyde solution, embedded in paraffin, cut into 2 μm sections, stained (hematoxylin–eosin staining, H&E), and examined with a Nikon Eclipse 50i microscope with Plan 10×, 40×, and 100× oil objectives. Images were captured and analyzed using the Nikon Digital Sight Ds-Fi1 high resolution digital camera (Nikon Instruments Inc., Tokyo, Japan) attached to the ocular tube.

### 3.2. Hardware and Software

All AI algorithms and feature extractions were run on a PC with Apple Mac Studio (2022), Apple M1 Max, 32 GB, a 1 TB SSD, and a 32-core GPU, Apple, Cupertino, California, USA.

The training and testing of AI algorithms in the software environment utilized Python (3.11) for PyCaret AutoML (3.0.4) and Python (3.12) for the CNN along with the following libraries: scikit-learn (1.5.2), TensorFlow (2.18.0), PyCaret (2.3.10), Keras (3.6.0), and Visualkeras (0.1.4.3).

The second-order features were extracted by using MATLAB 2021a (The MathWorks, Natick, MA, USA) and Image Processing Library.

### 3.3. Histopathological Image Dataset and Augmentation

The size of the acquired images was 1280 × 960 (width and height) in JPEG format.

To enhance the diversity of the training data and prevent overfitting, we employed three data augmentation strategies:

(i) Random rotations and affine transformations. The images were manipulated with random rotations of up to 10 degrees, and affine transformations were employed to implement scaling and translations, in order to enhance the model’s robustness to spatial transformation;
(ii) Random zoom. This creates surrounding pixel values in the image or interpolates pixel values according to the established percent; in this case, 30% was proposed;
(iii) Random horizontal and vertical flips. These were applied with probabilities of 30% for the horizontal orientation [37].

### 3.4. PyCaret AutoML

A machine learning algorithm is a method that processes data to identify a specific hypothesis from a set of candidates, optimizing performance in accurately representing the target function based on training experience. Numerous machine learning algorithms have been developed for various purposes; they differ in how they represent the hypothesis set, the approach to hypothesis selection, and the interaction of the model with the training data [38].

All textural features were imported into the PyCaret tool. It is an open-source, low-code machine learning framework in Python that optimizes specific parameters. PyCaret is frequently utilized in classifying features [39,40] due to its provision of a simplified and efficient process for comprehensive machine learning. It automates numerous activities, including data pretreatment, model selection, feature engineering, and hyperparameter tuning, therefore diminishing the necessity for manual intervention. Fivefold cross-validation enabled the optimization of the five highest-performing models selected from the following ML algorithms: KNN, Extra Trees Classifier (ETC), Logistic Regression, Ridge Classifier, RF, Classifier Light, Ada Boost Classifier, SVM, Linear Kernel Gradient, Naive Bayes, Decision Tree Classifier, Quadratic Discriminant Analysis, Dummy Classifier, Gradient Boosting Machine, Linear Discriminant Analysis (LDA), and Boosting Classifier. Subsequently, we created a random partition of the dataset (80% allocated for training and validation and 20% designated for testing) [41].

### 3.5. Custom-Built CNN

A class of machine learning models known as “deep learning” models use multilayer representations to extract hierarchical features from complex inputs. CNNs, recurrent neural networks (RNNs), generative adversarial networks (GANs), and deep belief networks are examples of common deep learning models that are based on neural network architectures [38].

**Figure 1:**
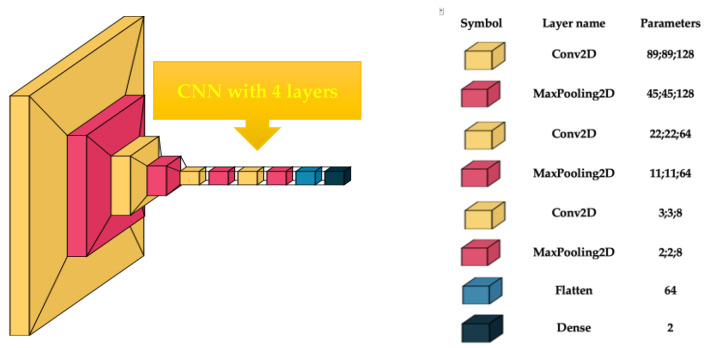
Custom-built CNN architecture.

This endeavor aimed to accurately analyze gynecologic histopathological images through the use of custom-built models. With an ablation process, the hyperparameters of the CNN were established. The image size was between 50 × 50 and 200 × 200, with a batch size of 4, 8, and 16, and epochs of 10 to 50. The optimum values for hyperparameters when a higher accuracy was obtained were an image size of 180 × 180 pixels, a batch size of 4, and 20 epochs. The customized CNN comprised four convolutional blocks, and the parameters of each layer are shown in Figure 1. Conv2D comprises a convolutional layer succeeded by batch normalization to enhance learning stability by normalizing the input to each layer. The ReLU activation function was utilized to incorporate nonlinearity. MaxPooling layers were incorporated following each convolutional block to diminish the spatial dimensions by fifty percent, preserving just the most salient information. Flattening was employed to transform all resultant two-dimensional arrays from pooled feature maps into a singular, extended linear vector. A dense layer was identified by its neurons being interconnected with every neuron in the preceding layer.

### 3.6. Co-Occurrence Matrix

A Gray-Level Co-occurrence Matrix (GLCM) provided an excellent foundation for image analysis and has proved to be important in many classification tasks. The contrast, entropy, energy, and homogeneity features are popular approaches to investigating image textures. The two most essential GLCM components are direction and distance. The horizontal, right, vertical, and left diagonal directions are the four possible direction angles for a two-dimensional image (0°, 45°, 90°, and 135°) [15]. The distance value can be adjusted to any larger number in order to determine the correlation between distant pixels, or it can be set to 1 for the pixel that is closest to its neighbour. Given that N is the total number of gray levels and is the component at the location in the normalized symmetrical GLCM, the following features are given [42].

The contrast measures the disparity or Intensity difference between a pixel and Its surrounding area over the full image.

(1) $$Contrast=\sum_{i,j=0}^{N-1}S_{ij}{\left(i-j\right)}^{2}$$

The degree to which the distribution of GLCM elements closely resembles the GLCM diagonal is known as homogeneity.

(2) $$Homogeneity=\sum_{i,j=0}^{N-1}\frac{S_{ij}}{1+{\left(i-j\right)}^{2}}$$

Energy is calculated by summing squared components.

(3) $$Energy=\sum_{i,j=0}^{N-1}{\left(S_{ij}\right)}^{2}$$

Entropy is a measure of an image’s randomness; it is low for smooth images and high for non-texturally uniform images [30].

(4) $$Entopy=-\sum_{i,j=0}^{N-1}S_{ij}logS_{ij}$$

where $S_{ij}$ is the probability of the value pair i and j.

### 3.7. Performance Evaluation Metrics

In our experiment, we employed an AutoML model with fifteen classifiers and a custom-built CNN. To accurately evaluate the proposed model, we employed a 5-fold cross-validation method. The confusion matrix produced in a classification process is a 2 × 2 matrix that indicates the model’s performance as a classifier for the test set with known actual target labels. The outcomes of the suggested forgery detection are presented in the confusion matrix as true positives (TPs), true negatives (TNs), false positives (FPs), and false negatives (FNs).

The accuracy is the proportion of accurately predicted occurrences to the total number of instances in the dataset, and the F1-score is a measure of predictive performance. These principal metrics are given by Equations (5)–(7) [43].

Accuracy = (TP + TN)/(TP + TN + FP + FN)(5)

F1-score = (2∙TP)/(2∙TP + FP + FN)(6)

(7) $$\mathrm{AUC}=1-1/2\;\left(\mathrm{FP}/\left(\mathrm{FP}+\mathrm{TN}\right)+\mathrm{FN}/\left(\mathrm{FN}+\mathrm{TP}\right)\right)$$

## 4. Results and Discussion

### Histopathological Image Dataset and Augmentation

**Figure 2:**
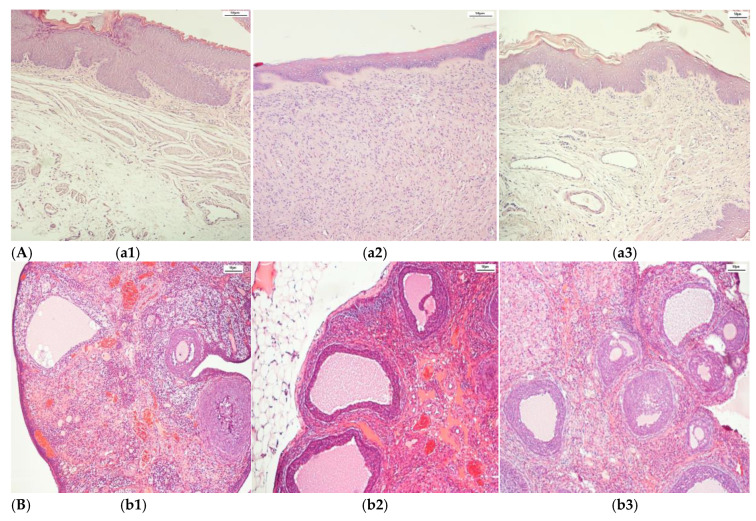

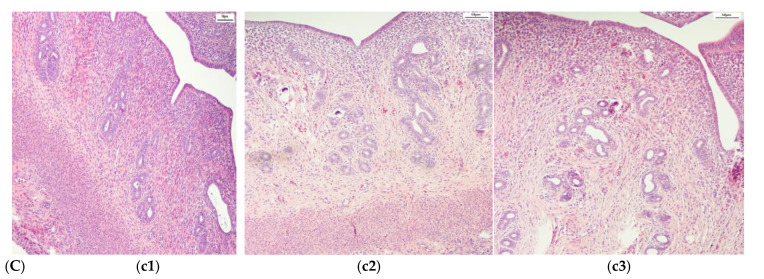
Samples of histopathological images. (**A**). Vagina (**a1**) Control, (**a2**) DM, and (**a3**) DM + synthetic compound; (**B**). Ovary (**b1**) Control, (**b2**) DM, and (**b3**) DM + synthetic compound; (**C**) Uterus (**c1**) Control, (**c2**) DM, and (**c3**) DM + synthetic compound.

**Figure 3:**
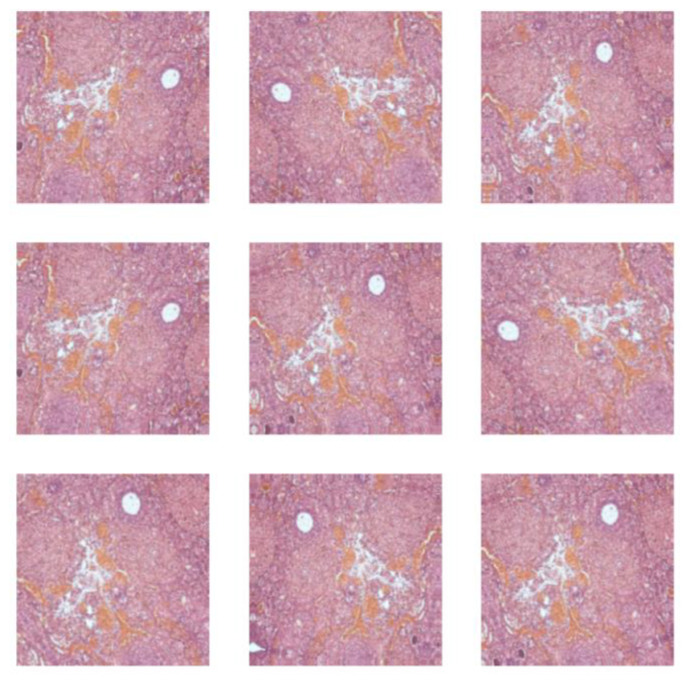
Augmented histopathological images.

The complex dataset after an augmentation process contained histopathological images of adult Wistar female rat (280 g weight) vaginal tissue (control (216), alloxan-induced diabetes mellitus (405), and diabetes mellitus and antidiabetic therapy with a synthetic compound (AD_SC) (603), uterine tissue (control (288), alloxan-induced diabetes mellitus (270), and diabetes mellitus and antidiabetic therapy with a synthetic compound (AD_SC) (612)), and ovary tissue (control (333), alloxan-induced diabetes mellitus (666), and diabetes mellitus and antidiabetic therapy with a synthetic compound (AD_SC) (360)). Samples from each class are shown in Figure 2. The augmented images are shown in Figure 3, after applying augmentation.

The data was randomly partitioned into 80% for training and validation and 20% for testing. We employed fivefold cross-validation on the training and validation sets (80%) using AutoML PyCaret for classification of textural features for the control and DM vagina, healthy and DM uterus, and control and DM ovary histopathological image classes. The experiment was repeated for the control and AD_DC vaginal, healthy and AD_DC uterine, and healthy and AD_DC ovarian histopathological image classes. Using the same number of classes, and keeping the same percentages for the training and testing sets, the custom-built CNN was trained. Hyperparameters were carefully optimized during training to guarantee the selection of the most effective model for the studied classes. Table 2 shows the results obtained with the proposed CNN. The performance of the model is expressed in terms of accuracy and F1-score. In addition, the confusion matrices are presented in the last column. Table 3 shows a detailed comparison of the performance of the top machine learning models fed with textural features via fivefold cross-validation on the test set, elucidating their strengths. In addition, the parameters of each ML classifier are shown. Following a comprehensive evaluation of AutoML models facilitated by the PyCaret tool, we discerned four models (ETC, RF, KNN, and LDA).

The noteworthy accuracy of 0.945 achieved by our custom-built model for uterine classes outshines that of the models proposed by Kitaya et al. (92.8%), Song et al. [8] (91.7%), Zhao et al. [9] (95.34%), Li et al. [10] (75%), and Volinsky-Fremond et al. [14] (78.9%). Also, the best accuracy was obtained in comparison with the pre-trained models proposed by Peng et al. [12] (86% AUC), Kodipalli [22] (67%), and Kwatra and Kaur [23] (92.8%).

Additionally, textural features were extracted from histopathological images in the diabetes mellitus and antidiabetic therapy with a synthetic compound class for the uterine, ovarian, and vaginal organs. We verified the results obtained with the PyCaret tool. The results demonstrate high performance for the antidiabetic therapy vagina class with an accuracy of 0.86 and the validation of the model with an F1-score of 0.88. In this case, Linear Discriminant Analysis was selected by AutoML as the best classifier.

Initially, the effectiveness of our approach may fluctuate when utilized on a large dataset, and AutoMLs are fed a large number of features. The extrapolation of our results to alternative datasets, especially with other diabetes classes, necessitates additional examination. Secondly, despite employing stringent augmentation methods to improve model robustness, the small number of rats from which the images were acquired and the existence of noise or artifacts in the input data may have adversely affected the model performance.

The limitations of this study include the high computational power required. In accordance with the hardware architecture, the time consumed exceeded two minutes. This was due to the use of CNNs and the limited number of histopathological images, as the dataset was originally based on a small number of rats. The use of pre-trained CNNs was not feasible due to the very low accuracy of the obtained results.

## 5. Conclusions and Future Scope

An important advancement in medical pathology is the use of digital or digitalized histopathology images for the identification of genital pathology for DS and AD_DC tissues. Additionally, because ML and CNN approaches may identify numerous unexplored regions, they have opened up new study prospects.

We consider that the high degree of interest in the research area related to diabetes mellitus and its effects on the female reproductive system supports more and more the use of digital or digitalized histopathologic images. Additionally, because ML and CNN approaches may identify numerous unexplored regions, they have opened up new study prospects.

This study introduced a novel model (a custom-built CNN for predicting three types of gynecological tissue (vaginal, uterine, and ovarian)) and, in parallel, classified textural features extracted from vaginal, uterine, and ovarian tissue using the PyCaret AutoML tool. In the first part of the study, the proposed CNN was found to provide high classification accuracy for uterine DS tissue as well as uterine AD_DC tissue. The second part of the study evaluated the classification performance of PyCaret AutoML; in this case, the best selected classifier was LDA for vaginal AD_DC tissue. Future studies should investigate supplementary classification algorithms or alternative preprocessing strategies to increase the accuracy of classification. Moreover, the authors propose to increase the number of images acquired in the laboratory to create a large dataset of histopathological images, classified by clinicians into subsets, as a valuable resource for researchers. This dataset could be used in designing experimental animal models, allowing for more extensive studies on the impact of diabetes mellitus on the female reproductive system.

## Figures and Tables

**Table 1:** Comparison of DL models for gynecological tissue.

References/Year	Weakness	AI Algorithms	Results Accuracy (%)
Patil and Patil [4]/2025	-focuses on enhancing a CNN type -time-consuming	TL-CNN	89.59%
Sun et al. [5]/2019	-low accuracy -a limited ability to model	CNN	84.50%
Kitaya et al. [6]/2024	-custom-built CNN	CNN	92.8%
Asadpour et al. [7]/2024	-focuses on using a pre-trained CNN -low accuracy	ResNet-50	82.88%
Song et al. [8]/2022	-only a custom-built DNN	DNN	91.7%
Zhao et al. [9]/2022	-global-to-local multi-scale CNN (G2LNet)	CNN	95.34%
Li et al. [10]/2023	-only a custom-built DNN -low accuracy	DNN	75%
Zafar et al. [11]/2025	-ML uterine tissue highly correlated 65 features.	Gated Highway Multi-layer-perceptron (GHiM)	86.6%
Peng et al. [12]/2020	-only a pre-trained CNN	ResNet V2	86% (AUC)
Onishi et al. [13]/2022	-low accuracy -without fine-tuning	10 conventional ML	84%
Volinsky-Fremond et al. [14]/2024	-low accuracy	Multimodal DNN	78.9%
Jeleń et al. [15]/2025	-without fine-tuning	NN, SVM	93.3%
Jagendra et al. [16]/2024	-only a neural network and a pre-trained CNN	VGG 16, Recurrent neural network (RNN)	94.65% 87.65%
Rajan et al. [17]/2024	-time-consuming	Inception v3, ResNet, XGBoost, SVM, KNN	90.37%
Zhou et al. [18]/2024	-time-consuming	SVM, RFXGBoost, CNN	94.6%
El-Latif et al. [19]/2024	-only a pre-trained CNN	ResNet-5	98.99%
Wang et al. [20]/2022	-low accuracy	InceptionV3, ViT	76%
Behera et al. [21]/2024	-only a pre-trained CNN -low accuracy	EfficientNet-B0	78%
Kodipalli [22]/2022	-only a pre-trained CNN -low accuracy	ResNet v2	67%
Kwatra and Kaur [23]/2023	-pre-trained -time-consuming	MobileNetV3 ResNet50	96.3% 92.08%
Radhakrishnan et al. [24]/2024	-time-consuming	MobileNetV2, VGG19, ResNet18, ResNeXt, Xception, EfficientNet	97.96%

**Table 2:** Comparison of the best performances of the CNN algorithm.

Classes	Test Set	Accuracy	F1-Score	AUC	Confusion Matrices [[TP FP][FN TN]]
Ovarian DC	133	0.842	0.857	0.842	[[63 9][12 49]]
Uterine DC	55	0.945	0.950	0.943	[[29 2][1 23]]
Vaginas DC	62	0.75	0.75	0.739	[[26 8][8 20]]
Ovarian AD_DC	44	0.771	0.733	0.736	[[22 5][6 11]]
Uterine AD_DC	92	0.858	0.853	0.858	[[38 7][6 41]]
Vaginas AD_DC	82	0.771	0.853	0.786	[[37 6][12 27]]

**Table 3:** Comparison of the best performances of PyCaret AutoML.

Classes	Accuracy	F1-Score	The Selected Classifier
Ovarian DC	0.785	0.837	ExtraTreesClassifier(criterion = ‘gini’, estimators = 100, random_state = 123)
Uterine DC	0.615	0.573	KNeighborsClassifier(algorithm = ‘auto’, leaf_size = 30, metric = ‘minkowski’, n_neighbors = 5)
Vaginas DC	0.775	0.788	LinearDiscriminantAnalysis(solver = ‘svd’, tol = 0.0001)
Ovarian AD_DC	0.751	0.822	RandomForestClassifier(criterion = ‘gini’, n_estimators = 100)
Uterine AD_DC	0.7	0.53	LinearDiscriminantAnalysis(solver = ‘svd’, tol = 0.0001)
Vaginas AD_DC	0.86	0.88	LinearDiscriminantAnalysis (solver = ‘svd’, tol = 0.0001)

## Data Availability

The original contributions presented in this study are included in the article. Further inquiries can be directed to the corresponding authors.

## Abbreviations

The following abbreviations are used in this manuscript:

DM	diabetes mellitus
AD-SC	antidiabetic therapy with a synthetic compound
CB-CNN	custom-built convolutional neural network
AutoML	PyCaret Auto Machine Learning
LDA	Linear Discriminant Analysis
DL	deep learning
ML	machine learning
AI	artificial intelligence
PCOS	polycystic ovary syndrome
XGBoost	extreme gradient boosting
KNN	k-nearest neighbor
RESNET	residual networks
RF	random forest
